# Impact of intra- and interfractional motion on the dose distribution and urinary adverse events for magnetic resonance-guided prostate cancer radiotherapy

**DOI:** 10.1016/j.phro.2026.100941

**Published:** 2026-03-03

**Authors:** Shu Xing, Sarah Burleson, Xiao Tan, Qijie Huang, Nima Hassan-Razein, Chengcheng Gui, Ergys Subashi, Himanshu Nagar, Victoria Brennan, Neelam Tyagi

**Affiliations:** a Department of Medical Physics, Memorial Sloan Kettering Cancer Center, New York, NY, United States; b Department of Radiation Oncology, Memorial Sloan Kettering Cancer Center, New York, NY, United States; c Department of Radiation Physics, Division of Radiation Oncology, University of Texas MD Anderson Cancer Center, Houston, TX, United States

**Keywords:** Prostate cancer, Intrafraction motion, Dose-blurring, Urinary adverse events

## Abstract

**Purpose:**

To evaluate the impact of intra- and interfractional motion on dose distribution and clinical outcome in prostate magnetic resonance (MR)-guided adaptive radiotherapy.

**Methods:**

Seventy-six patients were treated with five-fraction stereotactic radiotherapy on a 1.5 T MR linear accelerator. Cine MR images were acquired during treatment and exported to a motion monitoring research package, to generate motion traces in the anterior-posterior (AP), superior-inferior (SI) and left–right (LR) directions. Planned dose distributions were shifted according to motion trace to reconstruct intrafraction motion-blurred dose. Intrafraction motion-blurred doses were deformably registered to fraction one motion-blurred dose to obtain interfraction accumulated dose. The impact of motion on prostate, bladder, rectal and urethral dose-volume parameters were assessed. Correlation between patients experiencing prolonged motion > 3 mm and urinary adverse events was evaluated.

**Results:**

The average intrafraction prostate motion was −0.0 ± 0.2 mm (LR), 0.3 ± 0.5 mm (AP), and −0.3 ± 0.6 mm (SI). Intrafraction motions were associated with dose-volume parameter deviations up to 2.5 Gy. During 24 months of follow-up, 3 patients experienced ≥ Grade 2 acute adverse events, and 4 patients reported ≥ Grade 2 long term adverse events evaluated with common terminology criteria for adverse events. All patients with clinically significant urinary adverse events experienced prolonged large intrafraction motion > 3 mm. Interfraction accumulated motion-blurred dose demonstrated substantial interpatient variabilities for bladder, rectum and urethra.

**Conclusion:**

Prolonged intrafraction motion > 3 mm may result in dose difference and clinically significant urinary adverse events. However, combined intra- and interfraction motion might average out fraction-specific variations.

## Highlights

• Most prostate treatment fractions (>75%) experienced intrafraction motion < 3 mm.
• Intrafraction motion resulted in a maximum dose-volume parameter deviation of 2.5 Gy.
• All patients with urinary adverse events experienced prolonged motion > 3 mm.

## Graphical abstract

**Figure:**
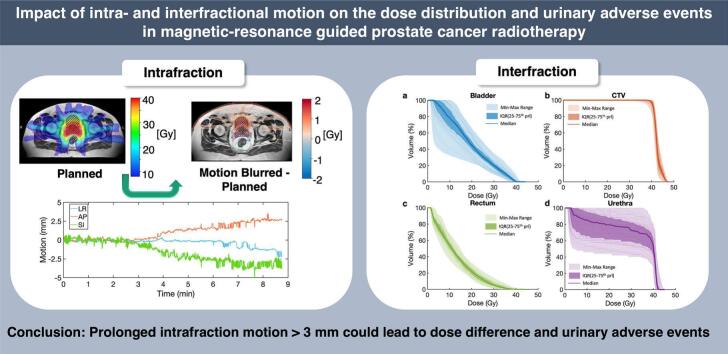

## 1 Introduction

Prostate cancer is the most commonly diagnosed cancer in men worldwide, with more than 1.4 million cases and approximately 400,000 deaths in 2022 [1]. External beam radiotherapy is one of the major treatment modalities for prostate cancer [2]. A recent clinical trial (PACE-B) demonstrated that ultra-fractionated stereotactic body radiotherapy treatment (SBRT) resulted in non-inferior clinical outcomes and adverse events compared to conventional and moderate fractionation [3]. As a result, SBRT treatment has gained popularity, as it significantly shortens the treatment course and reduces the number of visits for patients. With increased radiation dose delivered per fraction, the management of prostate motion during treatment becomes critical for achieving optimal tumor targeting while minimizing dose to organs at risk (OARs). The recent development of cone beam computed tomography (CBCT) − or magnetic resonance (MR) − guided online adaptive radiotherapy has reduced the uncertainties associated with interfraction prostate motion. However, understanding the intrafraction prostate motion remains a challenge.

Different techniques including fiducial tracking with X-ray imaging and transponders detection with non-ionizing electromagnetic localization system have been used to study intrafraction prostate motion. Levin-Epstein et al. investigated prostate motion for 205 SBRT patients, by using kilovoltage planar imaging to track the displacements of intraprostatic fiducials [4]. They observed that 2% and 5.4% of patients experienced greater than 3 mm motion in the superior-inferior (SI) and anterior-posterior (AP) directions, respectively. In addition, several studies have monitored prostate motion in real-time, using detected resonant response from implanted transponders [5], [6], [7]. The average motion ranged from 0.1 mm in the lateral direction to 6.3 mm in the longitudinal direction [5]. While these techniques offer valuable insights to intrafractional motion, they assumed that the movement of the fiducials accurately represented the motion of the prostate.

MR-guided adaptive radiotherapy (MRgRT) systems offer unique advantages for prostate SBRT. By providing superior visualization of the prostate, MRgRT allows for daily plan adaptation to account for geometric and anatomic changes. Moreover, due to the lack of ionizing radiation, continuous cine imaging can be used for real-time assessment of prostate motion, instead of using a surrogate [8]. Several centers have studied intra- and interfraction prostate motion with MR-Linac systems, which have shown improved target coverage, and reduced adverse events through margin reduction [9], [10], [11]. In the Phase III MIRAGE trial, MRgRT based SBRT [12] showed lower incidence of acute urinary and gastrointestinal adverse events than CT based SBRT.

Despite these benefits, the online adaptive workflow can lead to extended treatment times, which may increase the likelihood of intrafraction prostate motion. Multiple studies have reported correlations between prolonged treatment times and increased prostate displacement, with shifts up to 2 mm for treatments exceeding 15 min [13], [14]. Given these findings, understanding the clinical implications of inter- and intrafractional motion during MRgRT is of great interest. Our study provides a comprehensive analysis of intrafraction motion for SBRT patients with dose escalation on a 1.5 T MR-linac system. The effect of inter- and intrafractional motion on dose-volume parameters and its impact on clinical outcomes was also investigated.

## 2 Materials and methods

### 2.1 Patient data

This retrospective study included seventy-six prostate SBRT patients, treated with Unity MR-linac system (Elekta, Sweden) from 2020 to 2022. The study was approved by our institution’s Internal Review Board (IRB #21–308 and IRB #21–129). Sixty-six patients received 40 Gy to the prostate with a boost of 45 Gy to the dominant intraprostatic lesion (DIL). Ten patients received re-irradiation at lower prescription dose (25–36 Gy). A margin of 3 mm around the clinical target volume (CTV) was used to generate the planning target volume (PTV). A standardized bladder and rectum preparation protocol was strictly followed prior to treatment to ensure an empty bladder and rectum during treatment.

### 2.2 Patient treatment workflow

The treatment workflow is illustrated in Fig. S1. For each fraction, pre-treatment 3D T2-weighted (T2w) images were acquired using a turbo spin echo sequence. The adapt-to-shape workflow was implemented to generate an adapted plan that accounted for daily anatomical variations. During treatment, three orthogonal plane 2D cine MR images intersecting the prostate were acquired at 5 frames/s. The treatment was delivered, while monitoring the cine images and manually holding the beam if the prostate drifted out of the PTV. The detailed clinical workflow was described in Supplementary material A and in Brennan et al. [15]. The cine images were exported to a motion monitoring research package (MMRP), a precursor to the current comprehensive motion management system from Elekta. The first 90 frames were used to create a template by rigidly registering the 2D cine images to the 3D T2w MRI. The subsequent images were registered to the template, generating a motion trace that captured the center of mass shift of the target in the AP, SI and LR directions [16].

### 2.3 Intrafraction motion analysis

The average and maximum motion along the AP, SI and LR directions were analyzed for all treatment fractions. Histogram distributions of the average and maximum motion were fitted with either a unimodal or bimodal Gaussian function. The motion traces were post processed with a low-pass moving average filter using a 5-second window to reduce signal noise. At a given time, the probability of motion exceeding 1 mm, 2 mm, and 3 mm was estimated by calculating the percentage of treatment fractions in which the motion exceeded these thresholds. The analysis was conducted over a 10-min time range, at 30 s intervals. Additionally, we evaluated the frequency of prolonged large motion, which is considered as motion exceeding 3 mm for more than 10% of the treatment time. Treatment time was defined as the interval from the start of beam delivery until the end of beam delivery. Histograms were generated depicting the number of treatment fractions in which motion fell within 1–2 mm, 2–3 mm and > 3 mm for 0–10% (0 excluded) and > 10% of the treatment time. The 10% threshold was selected to differentiate transient and prolonged motion, as transient motion may have minimal impact on the total dose. A percentage-based threshold was chosen rather than an absolute time cutoff to remove dependence on the total treatment time.

### 2.4 Dose-volume parameter assessment from Intra- and Interfration motion

**Fig. 1:**
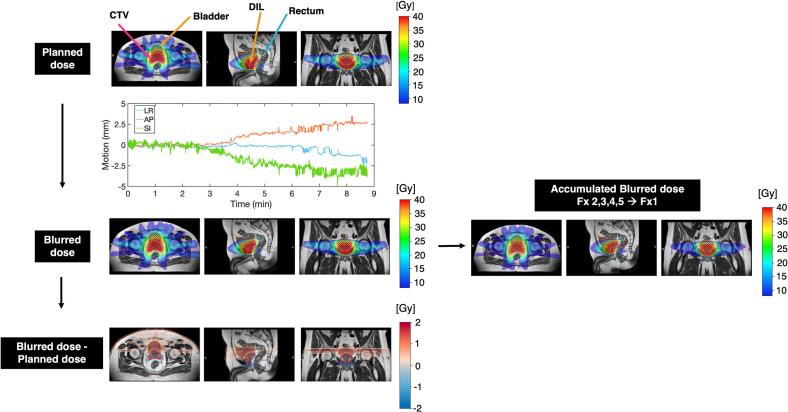
The dose reconstruction workflow to generate the motion-blurred dose distribution is demonstrated. Planned dose distributions for the clinical tumor volume (CTV), dominant intraprostatic lesion (DIL), bladder and rectum are shown for a representative patient. The daily planned dose distribution was shifted in all three directions based on the recorded motion traces over the treatment time. The adapted plan was optimized to the full prescription dose, although only the fractional dose was delivered. The cumulative shifted dose distributions were averaged over all frames to produce a motion-blurred dose distribution. The difference between the planned and motion blurred dose distributions was then computed and displayed with a diverging colormap centered at 0. Dose difference < 1% of the prescription dose was masked (white). In addition, the motion-blurred dose was also accumulated over five fractions through deformable registration.

To quantify the impact of intrafraction motion on delivered dose, a motion-blurred dose distribution was reconstructed (Fig. 1). For each fraction, the daily adapted dose distribution was shifted according to the magnitude and direction of the motion trace at every 0.2 s. The cumulative shifted dose distributions were averaged over time to produce a motion-blurred dose distribution. The difference between the planned and motion-blurred dose distributions was calculated.

The accumulated motion-blurred dose across five fractions was reconstructed using a customized MIM Maestro workflow (MIM software Inc., Beachwood, OH, USA). The pre-treatment MRI of all subsequent fractions was rigidly registered to the pre-treatment MRI of the first fraction. Multi-modality deformable registration in MIM, which employs high-dimensional feature descriptors to maximize correspondence between images, was then applied to deform the pre-treatment MRIs from all subsequent fractions to that of the first fraction. The resulting deformation vector fields were used to deform the fractional motion-blurred dose distributions from fraction two through five. The deformed fractional motion-blurred doses were then combined using trilinear interpolation to obtain the accumulated dose, thereby accounting for both intra- and interfraction motion.

The CTV, bladder, rectum and urethra dose-volume histograms (DVHs) from the planned, motion-blurred and interfraction accumulated motion-blurred dose distributions were computed for all patients. The median, inter-quartile range (IQR) and the minimum-to-maximum range of the DVHs from the accumulated dose were determined. Clinically relevant dose parameters were also evaluated, including dose to 95% of the CTV (D_95%, CTV_), along with key urinary adverse events predictors such as maximum dose to the urethra (D_max,urethra_) and bladder (D_max,bladder_). Dose received by 10% (D_10%, bladder_) and 50% (D_50%, bladder_) of the bladder volume, dose received by 10% (D_10%, urethra_) and 90% (D_90%, urethra_) of the urethra volume were also examined. Additionally, mean rectal dose (D_mean,rectum_) and rectal volume receiving at least 10 Gy (V_10Gy, rectum_), 24 Gy (V_24Gy, rectum_) and 30.15 Gy (V_30.15Gy, rectum_) were evaluated. Changes in these parameters due to different intrafraction motion magnitude were also assessed.

### 2.5 Correlation with urinary adverse events

All patients in this study were followed for 24 months post-treatment. Urinary adverse events including urinary frequency, retention, incontinence, and hematuria were evaluated using the Common terminology criteria for adverse events (CTCAE), with grades from 0 to 5 indicating increasing severity. CTCAE grades were collected both within 6 months (acute adverse events) and beyond 6 months (long-term adverse events) post-treatment, with ≥ Grade 2 considered clinically significant.

Patients exhibiting persistent large motion throughout the treatment course ( > 3 mm for average percentage > 2% of the treatment time over five fractions) were identified (Fig. S4) and separated into two groups: those who developed urinary adverse events and those without. Dose-volume histogram (DVH) parameters from the motion blurred dose distributions of each fraction were compared between the two groups to assess potential association between motion and urinary adverse events. Each fraction was planned to the full prescription dose. The non-parametric Mann-Whitney *U* test was performed, with a p-value < 0.05 considered statistically significant. Similarly, the DVH parameters from the accumulated dose distributions were also compared between the two groups. All analysis in this study was conducted in MATLAB (Mathworks, Natick MA).

## 3 Results

A total of 76 patients with 369 treatment fractions were evaluated. The cine imaging data for 11 fractions were corrupted, therefore not included in the analysis. Patient characteristics were summarized in Table 1. The signed mean ± standard deviation prostate motion was −0.0 ± 0.2 mm, 0.3 ± 0.5 mm and −0.3 ± 0.6 mm in the LR, AP and SI direction, respectively. The distributions of the average and maximum prostate motion were shown in Fig. S2. The DICOM coordinate system orientation was followed, with a positive value pointing to the left, posterior and superior direction.

**Table 1:** Clinical characteristics of patient cohort included in this study.

**Characteristics**	**Patient number (n = 76)**
Age at prostate cancer diagnosis (median, range)	72 (54–85)
Prostate Size	49.2 mL (14 – 118.7 mL)
Stage	
T1c	29
T2	2
T2a	4
Unspecified	41
PSA	7.4 (2 – 19.3)
Urinary adverse events	
Group A: No clinically significant acute urinary adverse events	
Initial irradiation	61
Re-irradiation	6
Group B: Moderate acute urinary adverse events	
Initial irradiation	5
Re-irradiation	4
Prescription Dose	40 Gy with 45 Gy boost to DILs, 25–36 Gy for re-irradiation
Radiotherapy technique	IMRT
Beam number	15

**Fig. 2:**
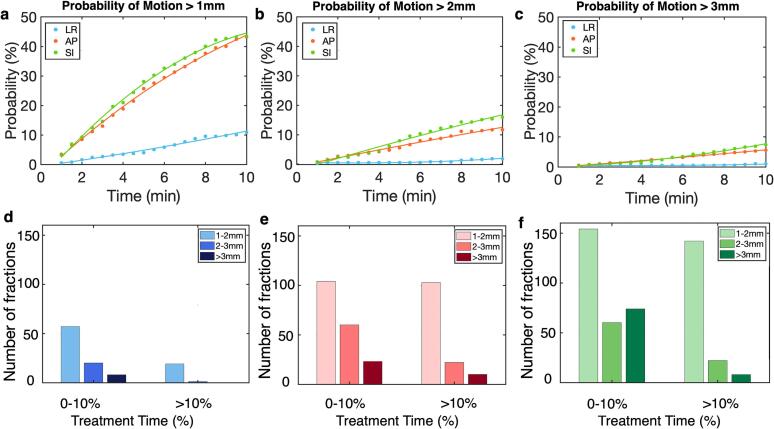
The probability of motion greater than 1 mm (a), 2 mm (b) and 3 mm (c) as time increases are demonstrated for motion in the left–right (blue), anterior-posterior (red) and inferior-superior (green) directions. Among the 369 total treatment fractions, the number of fractions with motion greater than 1, 2, and 3 mm for 0–10% (excluding 0%) and > 10% of treatment time are demonstrated, for motion in the left–right (d), anterior-posterior (e) and inferior-superior (f) directions. (For interpretation of the references to colour in this figure legend, the reader is referred to the web version of this article.)

The treatment time ranged from 5.2 min to 33 min, with an average of 9.0 ± 3.7 min. 24.4% of the fractions exceeded 10 min in treatment time. The probability of displacement increased over time, with a higher probability of displacement in the AP and SI directions (Fig. 2 a-c). At 10 min after treatment initiation, the probability of displacement exceeding 2 mm was at 18% in the SI direction and 12% in the AP direction, while the probability of motion exceeding 3 mm was at 8% and 9% in the AP and SI direction, respectively. Among the 369 treatment fractions, 19 fractions experience lateral motion exceeding 1 mm for over 10% of treatment time (Fig. 2d). In contrast, AP (Fig. 2e) and SI motion (Fig. 2f) showed more frequent displacements, with 22 fractions experiencing motion > 2 mm and 9 fractions exceeding 3 mm for > 10% of the treatment time. The SI direction demonstrated a higher tendency for prolonged motion between 1–2 mm, with 38% of the fractions fallen in this range, compared to 27% in the AP direction.

Most cases exhibited intrafraction prostate motion in the inferior and posterior direction, which resulted in increased dose to the bladder and reduced dose to the rectum. However, we observed the opposite trend for 30% of treatment fractions with prolonged large motion (example case shown in Fig. S3). Increased dose differences for bladder and rectal parameters were found to be associated with greater intrafraction motion. Table 2 presents the unsigned difference between fractional motion-blurred dose and daily planned dose for various DVH parameters, stratified by the extent of prostate motion during treatment. Little difference was found in D_95%, CTV_, D_max,urethra_ and D_max,bladder_. The mean difference ± standard deviation in D_10%, bladder_ increased from 0.5 ± 0.7 Gy in the 1–2 mm group to 2.1± 0.6 Gy in the > 3 mm group, whereas the difference in D_mean,rectum_ changed from 0.2 ± 0.4 Gy (1–2 mm group) to 1.0 ± 0.3 Gy (> 3 mm group). These differences were estimated based on the full prescription dose over all treatment fractions, therefore the per-fraction impact of intrafractional motion remained relatively small.

**Table 2:** Dose-volume histogram (DVH) parameter difference (unsigned) between motion-blurred and planned dose.

Unsigned DVH parameter difference (mean ± standard deviation) between motion-blurred and planned dose			
	Motion 1–2 mm for > 10% of treatment time	Motion 2–3 mm for > 10% of treatment time	Motion > 3 mm for > 10% of treatment time
D_95%, CTV_ (Gy)	0.1± 0.3	0.2± 0.2	0.1 ± 0.1
D_max,bladder_ (Gy)	0.2 ± 0.4	0.7 ± 0.6	0.7± 0.5
D_10%, bladder_ (Gy)	0.5 ± 0.7	1.6± 0.9	2.1 ±0.6
D_50%, bladder_ (Gy)	0.6 ± 1.0	2.0± 1.7	2.5 ± 2.1
D_mean,rectum_ (Gy)	0.2 ± 0.4	0.8 ± 0.4	1.0 ± 0.3
V_10Gy, rectum_ (mL)	0.2 ± 0.4	0.4± 0.4	0.8 ± 0.5
V_24Gy, rectum_ (mL)	0.1 ± 0.3	0.2 ± 0.5	0.4± 0.3
V_30.15Gy, rectum_ (mL)	0.6± 1.0	1.1± 1.4	2.4 ± 1.4
D_max,urethra_ (Gy)	0.2± 0.3	0.4± 0.6	0.4 ± 0.3

**Fig. 3:**
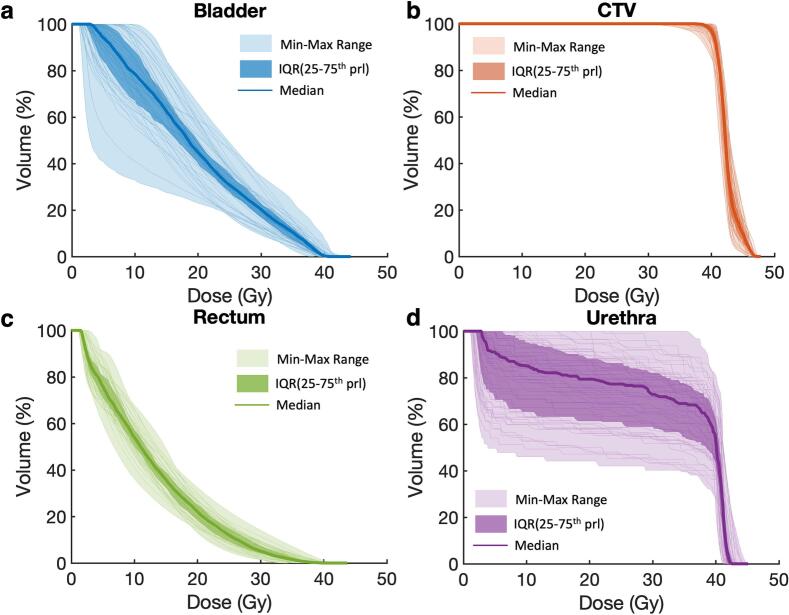
Dose-volume histograms (DVHs) derived from the interfraction accumulated motion-blurred dose distributions were plotted for (a) bladder, (b) clinical target volume (CTV), (c) rectum, and (d) urethra across all patients. For each structure, the shaded regions represent the minimum–maximum range (light shaded) and the interquartile range (dark shaded), while the solid line denotes the median DVH.

Fig. 3 overlayed the CTV and OAR DVHs derived from the accumulated motion-blurred dose distributions showing the effect of both inter- and intrafraction motion. The CTV coverage was well maintained, with D_95%, CTV_ = 40.4 Gy from the median DVH. D_max,bladder_ ranged from 38.3 Gy to 44.1 Gy, with a median (IQR) D_max,bladder_ of 41.2 (1.0) Gy. When considering clinical tolerance thresholds, 3% and 16% of patients exceeded the hard (43.5 Gy) and preferred (42 Gy) bladder dose constrains, respectively. The median (IQR) for D_max,urethra_ is 42.5 (1.0) Gy, ranging from 41.2 to 45.0 Gy. Based on the clinical constraint of 43.5 Gy, 6% of the patients exceeded the tolerance.

Nine patients (11.8%) reported ≥ Grade 2 urinary adverse events, among which four patients received re-irradiation (Table 1). Patients with re-irradiation were excluded from the investigation on the correlation between intrafraction motion and urinary adverse events, as re-irradiation is likely a confounding factor. For the remaining five patients, three patients experienced ≥ Grade 2 acute adverse events, and four reported ≥ Grade 2 long-term adverse events.

**Fig. 4:**
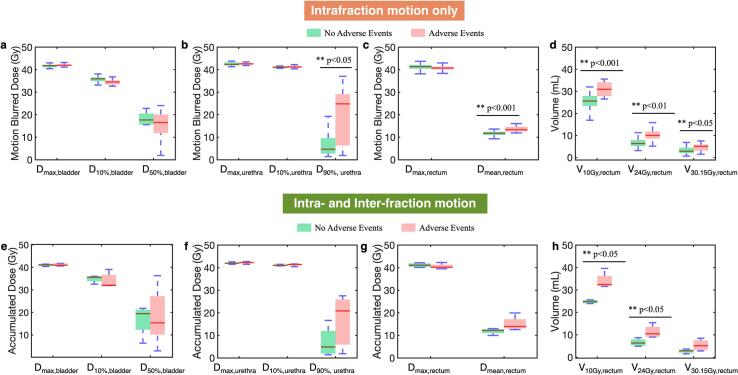
Dose-volume histogram (DVH) parameters from the motion blurred dose distributions were compared between the groups with and without adverse events for the bladder (a), urethra (b), and rectum (c,d). A p-value < 0.05 demonstrated statistically significant difference. The same DVH parameters computed from the motion-blurred dose accumulated over 5 fractions were also compared between the two groups (e-h).

A total of nine patients experienced prolonged large motion in the SI direction. The dose parameters of the motion-blurred dose were compared between the group with adverse events (5 patients) and the one without (4 patients). No significant differences were found in the bladder and most of the urethra dose parameters (Fig. 4a). However, patients with adverse events exhibited significantly (p < 0.05) higher D_90%, urethra_ (Fig. 4b). In addition, significantly higher D_mean,rectum_, V_10Gy, rectum_, V_24Gy, rectum_ and V_30.15Gy, rectum_ were observed in the adverse events group (Fig. 4 c, d). The dose parameters from the accumulated dose over the entire treatment were also compared between the group with adverse events and the one without (Fig. 4e-h). Significantly higher V_10Gy, rectum_, and V_24Gy, rectum_ were observed in the adverse events group.

## 4 Discussion

This work presented a comprehensive analysis of inter- and intrafraction prostate motion and its impact on DVH parameters. Intrafraction motion was generally small. Increased intrafraction motion was associated with greater deviations in bladder and rectal DVH parameters. Accumulated motion-blurred dose analyses revealed substantial interpatient variability in OAR doses with a subset of patients exceeding clinical bladder and urethral constraints. Notably, all patients with clinically significant urinary adverse events exhibited prolonged large intrafraction motion in the SI direction.

A notable tendency for posterior and inferior prostate motion was observed, aligning with previous findings from both cine-MRI-based and X-ray imaging-based studies [11], [17], [18], [19]. Prostate displacements also progressively increased with treatment duration. Although radiation therapists were instructed to manually pause treatment when motion exceeded 3 mm, motion beyond this threshold was still observed. In some cases, therapists waited to confirm that the prostate drift was sustained before interrupting the delivery, and this wait time was highly subjective. Similar trends were observed for patients with treatment times greater than 5 min [20], [21]. Langen et al. reported that the probability of 3D motion exceeding 3 mm progressively increased with treatment time, rising from 2% in the first minute to over 25% after 10 min [22]. The increased intrafraction motion of the prostate may be attributed to bladder or rectal filling, patient movement, or gradual relaxation of the pelvic floor musculature [23]. As the adaptive workflow with MRgRT has extended treatment time, intrafraction motion should be carefully considered.

The number of treatment fractions with prolonged large motion remained low, consistent with observations in previous studies [24], [25]. Minimal dose difference was observed in the CTV and the maximum dose to bladder and urethra. Similar observations have been reported in the literature, where a 2.6% dose difference in PTV coverage was noted between the planned and reconstructed dose with motion [26]. A 1 cm prostate shift was needed to result in a 14% decrease in prostate D95% dose [27]. Additionally, differences of less than 5% have been reported for rectal and bladder dose parameters [26], [28].

All patients who experienced Grade ≥ 2 urinary adverse events in our patient cohort exhibited prolonged large motion, suggesting an association between motion and urinary adverse events. Similar observations were made in the MIRAGE trial, which demonstrated that treatment fractions with large intrafraction motion were associated with increased adverse events [29]. There were four other patients with prolonged large motion, but without adverse events, suggesting that factors other than intrafraction motion could also contribute to adverse events. Several studies reported an association between the maximum bladder and urethra dose and acute urinary adverse events [30], [31]. However, in our study, no significant difference in maximum bladder and urethra dose parameters were found between the adverse events and non-adverse events groups. We observed significantly (p < 0.05) higher D_90%, urethra_ in the adverse events group, when only considering intrafractional motion. This effect seems to have averaged out over fractions, as no significant difference was found for D_90%, urethra_ from the accumulated motion-blurred dose.

Our study had several limitations. First, our patient cohort was relatively small, with 2.6% of treatment fractions experiencing prolonged large motion and 5 out of 76 patients experienced any urinary adverse events. The small cohort size limited the statistical power of our findings. Moreover, because patients in this cohort were treated in 2020–2022, gastrointestinal adverse events data and disease control endpoints were not yet available at the time of the analysis. A study including a larger patient population and more diverse adverse events profile would be beneficial to validate these observations. Secondly, our dose reconstruction from intrafraction motion of the prostate, assumed no interplay between prostate and OAR motions or deformation of the prostate and OARs due to bladder and rectal filling or gas pockets. In addition, our motion-blurred dose reconstruction workflow employed a simplified blurring algorithm that did not account for multi-leaf collimator (MLC) motion, beam angles, gantry rotation speed or dose rate [32]. Future studies could incorporate more advanced dose reconstruction methods using machine log-file and 3D cine imaging to improve estimation accuracy. Finally, in this study, a commercial deformable image registration algorithm was used to deform and accumulate doses. Although the multimodality deformable registration introduces uncertainties, prior studies have demonstrated reasonable performance for dose accumulation in prostate patients [33].

## CRediT authorship contribution statement

**Shu Xing:** Data curation, Formal analysis, Methodology, Visualization, Writing – original draft, Writing – review & editing. **Sarah Burleson:** Data curation, Writing – review & editing. **Xiao Tan:** Data curation, Formal analysis. **Qijie Huang:** Data curation, Formal analysis. **Nima Hassan-Razein:** Data curation, Writing – review & editing. **Chengcheng Gui:** Data curation, Methodology. **Ergys Subashi:** Methodology, Writing – review & editing. **Himanshu Nagar:** Writing – review & editing. **Victoria Brennan:** Conceptualization, Resources. **Neelam Tyagi:** Conceptualization, Resources, Supervision, Writing – review & editing.

## Declaration of competing interest

The authors declare that they have no known competing financial interests or personal relationships that could have appeared to influence the work reported in this paper.
