# Supplementary material for: Impact of intra- and interfractional motion on the dose distribution and urinary adverse events for magnetic resonance-guided prostate cancer radiotherapy
Source: Phys Imaging Radiat Oncol. 2026 Mar 3;38:100941. doi: 10.1016/j.phro.2026.100941 (PMC12997195; doi:10.1016/j.phro.2026.100941)
Supplement: Supplementary Data 1 [file mmc1.pdf]

**Supplementary Material**

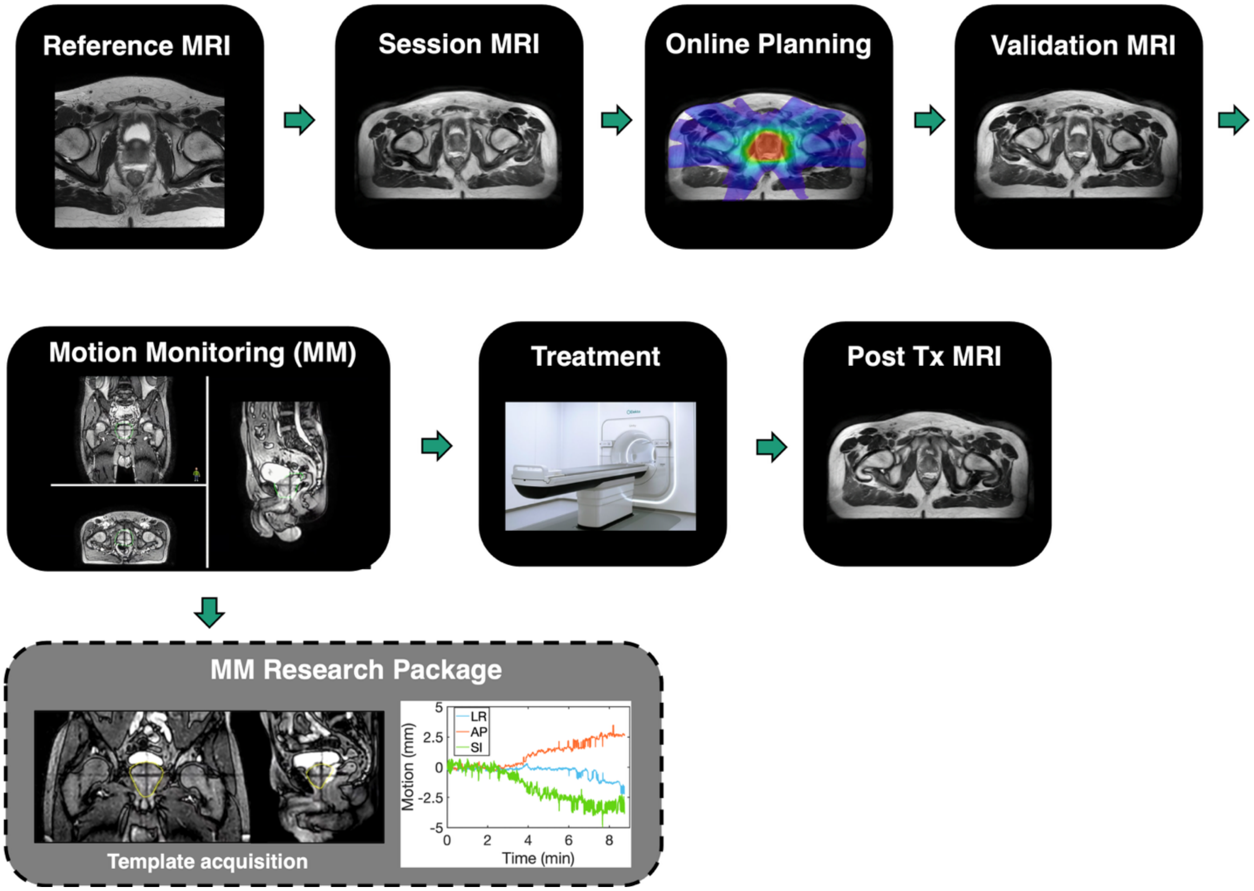


**Figure S1** Schematics of the clinical workflow used in this study. Motion monitoring system from Elekta was used to track the intrafraction motion of the prostate and manual beam hold was applied if the prostate drifted outside of the PTV during treatment. The motion management research package (MMRP) was used to generate the motion traces offline (in dotted line).

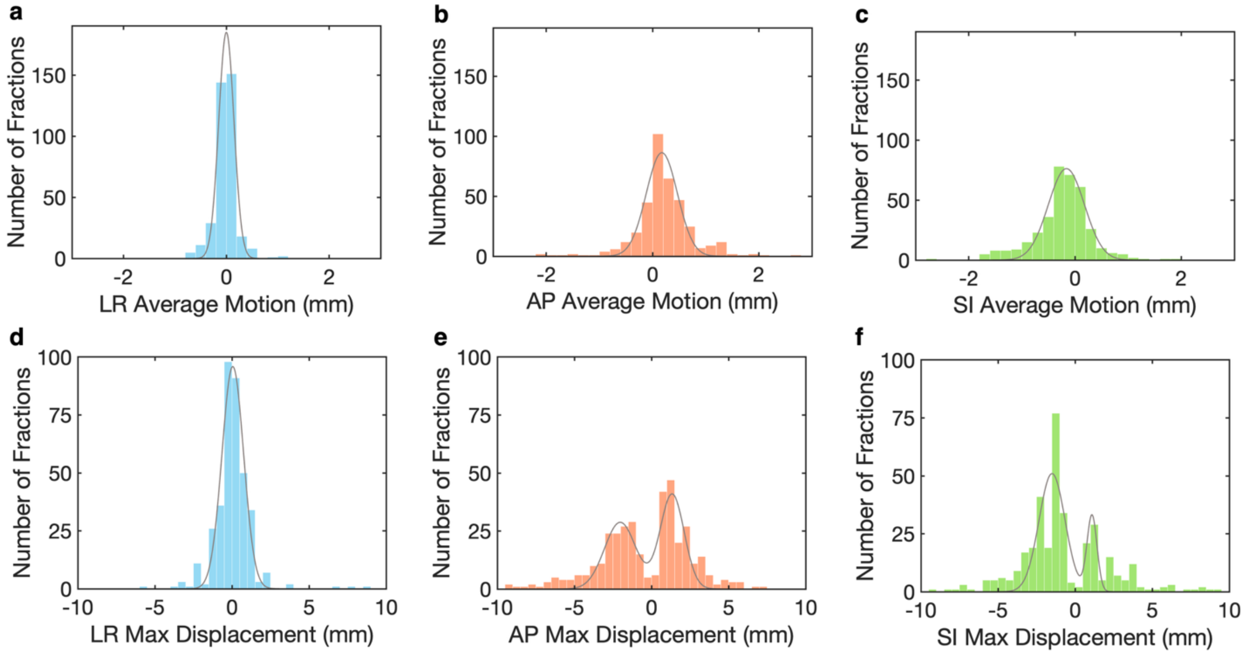


**Figure S2** Distributions of the average (a,b,c) and maximum (d,e,f) prostate motion along the left -right (LR), anterior-posterior (AP) and superior-inferior (SI) directions for each patient across all treatment fractions are demonstrated. A Gaussian distribution (a,b,c,d) or bimodal gaussian distribution (e,f) was fitted to the histograms to characterize the shape of the distribution.

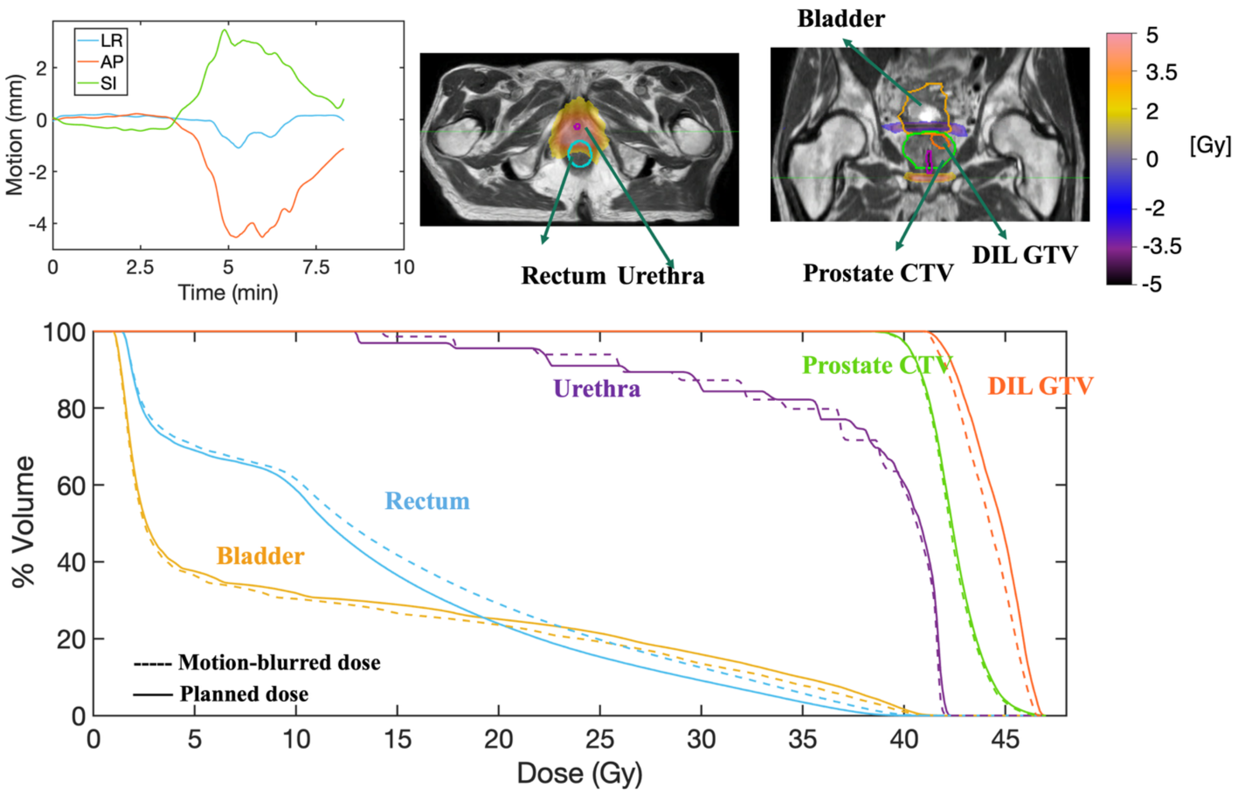


**Figure S3** An example patient with prolonged large intrafractional motion in the SI and AP directions was demonstrated. The dosimetric difference map were generated by subtracting the motion-blurred dose distribution from the daily planned dose distribution. In the dose-volume histogram (DVH) comparison, the dotted lines represent the DVHs from the motion blurred dose distribution while the solid line represent the daily adapted planned dose distribution.

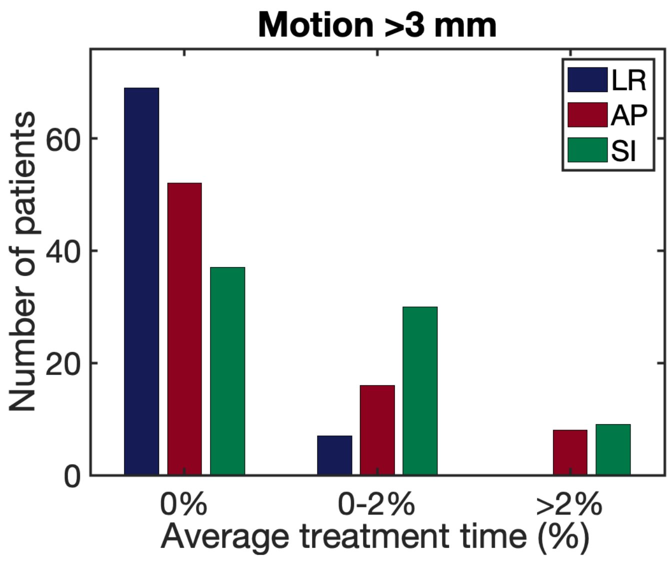


**Figure S4** The percentage treatment time was averaged over 5 fractions to assess the overall prolonged motion of the treatment. The number of patients that experienced motion > 3mm in the left-right (LR), anterior-posterior (AP) and superior-inferior (SI) for 0%, 0-2% (excluding 0%) and > 2% of average percentage treatment time was illustrated.

**Supplementary Material A**

The treatment workflow is illustrated in Figure S1. For each fraction, pre-treatment 3D T2-weighted (T2w) images were acquired using a turbo spin echo sequence with an echo time of 87 ms and a repetition time of 1300 ms. The voxel size was 1 x 1 x 2 mm3 with 400 x 450 x 250 mm3 field of view. The adapt-to-shape workflow was implemented to generate an adapted plan that accounted for daily anatomical variations. Before treatment, a verification 3D T2w MRI was acquired to correct for potential prostate shift due to bladder filling during the contouring and planning phase. During treatment, three orthogonal plane 2D cine MR images intersecting the prostate were acquired at 5 frames/s. The orthogonal cine MRI used a balanced turbo field echo sequence with a repetition time of 3.8 ms and an echo time of 1.92 ms. The acquisition voxel size was 3 x 3 x 5 mm3, whereas the reconstruction voxel size was 1.3x1.3x5 mm3, with 436 x 436 x 5 mm3 field of view. The treatment was delivered, while monitoring the cine images and manually holding the beam if the prostate drifted out of the PTV (CTV + 3 mm). A post-treatment MRI scan was also acquired to assess target and organ-at-risk (OAR) motion during treatment. The detailed clinical workflow was described in Brennan et al. [15]. The cine images of all treatment fractions were exported to a motion monitoring research package (MMRP), a precursor to the current

comprehensive motion management system from Elekta with automated gating capabilities. The first 90 frames were used to create a template by rigidly registering the 2D cine images to the 3D T2w MRI. The subsequent images were registered to the template, generating a motion trace that captured the center of mass shift of the target in the AP, SI and LR directions.

The average motion in all directions closely followed a Gaussian distribution ($R^2 > 0.98$), suggesting that prostate intrafraction motion adhered to a random walk model [16] (Figure S2 a-c). The mean ± standard deviation prostate motion was -0.0 ± 0.2 mm, 0.3 ± 0.5 mm and -0.3 ± 0.6 mm in the LR, AP and SI direction, respectively. The DICOM coordinate system orientation was followed, with a positive value pointing to the left, posterior and superior direction. The maximum motion distributions exhibited greater variability, ranging from -6.0 to 8.6 mm, -12.8 to 5.9 mm, and -15.6 to 33.8 mm in the LR, AP and SI directions, respectively. A bimodal pattern was observed in the AP and SI directions, suggesting a tendency for prostate displacement in the posterior and inferior directions. The LR maximum displacement remained symmetrically distributed around zero.

However, we observed the opposite trend for 30% of treatment fractions with prolonged large motion (> 3mm). We demonstrated an example patient case (Figure S3) with a displacement greater than 3mm in the anterior and superior direction for 22% (1.8 min) and 18% (1.5 min) of the treatment time, respectively. The resulting dose difference map, generated by subtracting the motion-blurred dose distribution from the daily planned dose distribution, revealed an increase in $D_{mean,rectum}$ by 1.25 Gy and a decrease in $D_{10\%,\ bladder}$ (Gy) of 1.6 Gy. The prostate CTV dose, maximum bladder and urethra dose remained largely unaffected.

The number of patients that experience motion > 3 mm in all three directions was evaluated (Figure S3). The percentage treatment time was averaged over 5 fractions, as toxicity was evaluated after the entire treatment course. 50% of the patients did not experience motion > 3 mm in any direction. Nine patients experienced motion > 3 mm for over 2% of the average percentage treatment time in the SI direction.
